# High Time for Pathology AI: Practical Considerations for Turning Potential into Practice

**DOI:** 10.3390/diagnostics16213653

**Published:** 2026-11-05

**Authors:** George M. Yousef, Elizabeth Attia, Mahtab Nazari, Maximilian Domann, Bahjat Hanna, Fatemeh Zabihollahy, Ioannis Prassas

**Affiliations:** 1 Laboratory Medicine Program, University Health Network, Toronto, ON M5G 2C4, Canadamaximilian.domann@uhn.ca (M.D.);; 2 Department of Laboratory Medicine and Pathobiology, University of Toronto, Toronto, ON M5S 1A8, Canada

**Keywords:** artificial intelligence, digital pathology, machine learning, precision oncology, validation, cost-effectiveness, time efficiency

## Abstract

Artificial intelligence (AI) has the potential to improve diagnostic accuracy, reproducibility, and efficiency in anatomical pathology, but technical success alone does not establish clinical utility. In this Perspective, we examine practical requirements for translating computational pathology tools into routine practice, including prioritization of genuine clinical bottlenecks, representative datasets, independent validation, workflow integration, costs, clinician oversight, regulatory compliance, and post-deployment monitoring. We distinguish evidence from retrospective model evaluations, controlled reader studies, prospective workflow assessments, and economic simulations, recognizing that their conclusions are not interchangeable. We also propose a staged, decision-oriented framework through which laboratories can assess clinical need, select technologies, perform local validation, conduct prospective pilots, deploy with safeguards, and monitor performance throughout the AI lifecycle. Evidence from selected applications is encouraging, but effectiveness, cost-effectiveness, and readiness for routine use vary by intended purpose and practice environment. Responsible adoption should therefore be guided by demonstrated clinical benefit, operational feasibility, and ongoing evaluation rather than algorithmic performance alone.

## 1. Introduction

As artificial intelligence (AI) makes its way into healthcare, and more specifically into pathology [1], the practical added value of implementing such technologies must be carefully considered. There are several important issues that need to be addressed, starting with assessing which are the specific pathology tasks in which AI can truly add value and where its most practical applications lie. Do these AI tools solve real clinical bottlenecks, or do they primarily serve tasks that are not truly rate-limiting in practice? How analytically reliable are they? What are the rates of error across time and what efficiency gains are associated with their implementation? How generalizable are these models across institutions and how can these systems be properly integrated into routine clinical practice while maintaining clinician trust? What is the true cost of using AI in pathology? The aim of this paper is to offer a careful assessment of the practical value of AI in pathology from a pathologist’s clinical perspective and to identify some key gaps that should need to be carefully considered before their widespread implementation in clinical practice.

This article is a selective, narrative Perspective, not a systematic or scoping review. We draw on illustrative diagnostic and reader studies, implementation reports, the methodological literature, and professional or regulatory guidance relevant to clinical translation. The references are used to examine practical decisions and limitations, not to provide an exhaustive evidence synthesis. No systematic search or meta-analysis was performed.

The purpose of this Perspective is to translate these familiar technical and organizational concerns into a practical decision process for pathologists, laboratory directors, and multidisciplinary implementation teams. We propose that adoption should proceed through explicit decision gates: clinical need, tool and evidence selection, local validation, prospective pilot assessment, controlled deployment, and lifecycle monitoring. This is a pragmatic framework synthesized from the considerations discussed here, not a formally validated standard. To make this decision process explicit, we also propose a readiness assessment by proposing a provisional multidomain readiness scorecard (Supplementary Table S2).

## 2. The Rapid Growth of AI in Pathology

A rapidly expanding number of AI algorithms for pathology applications are currently under development, both in industry and in academic centers. This expansion is evident in the surge of both academic publications and commercial products focused on AI-driven pathology tools. This momentum of innovation has been driven by several enabling factors. First, there is the adoption of whole-slide imaging (WSI) technologies in pathology departments. An increasing number of hospitals and pathology centers worldwide are getting equipped with digital scanners producing high-quality digital tissue images that are essential for algorithm training [2]. Coupled with this are the recent rapid advances in computational power and cloud infrastructure [3]. The availability of more affordable graphics processing units (GPUs) (note: they are still prohibitively costly for many departments) and the emergence of scalable computing ecosystems have enabled training and deployment of large neural networks [4]. In addition, the development of modern deep learning frameworks (PyTorch, TensorFlow) significantly lowered the technical barrier to building and scaling such models in pathology, while scalable cloud storage solutions removed some initial barriers related to local storage requirements (note: establishing sustainable long-term storage solutions remains an open challenge for many pathology departments). Crucially, these technical/infrastructural developments coincided with an unprecedented expansion in digital pathology data availability. Large image datasets from initiatives like The Cancer Genome Atlas (TCGA) and from open challenges (e.g., PANDA, CAMELYON) have promoted collaboration and have provided much-needed standardized, annotated WSIs for benchmarking and model training [5,6,7]. Lastly, the recent emergence of digital pathology foundational models pre-trained on millions of WSIs (summarized in Supplementary Table S1) further accelerated AI development in this field [8].

## 3. The Scope of Applications of Ai in Pathology

Digital pathology AI applications can be organized by intended function rather than by the computational method used to build them. Section 3 and Table 1 use the same five categories: image analysis and quantification; diagnostic detection and classification; prognostic and therapeutic decision support; workflow optimization; and exploratory analysis and hypothesis generation. The final category is primarily research-oriented and not an established clinical application. Categories may overlap, and the maturity of each tool must be assessed for its specific intended use, regulatory status, and available evidence.

▪ Image analysis and quantification

These applications identify tissue structures and provide quantitative measurements relevant to pathology assessment [9]. Examples include tumor region detection, mitotic counting, immunohistochemistry (IHC) scoring, fibrosis burden, and tumor cellularity assessment. Depending on the task, detection, segmentation, classification, or regression methods may be used. Their functional purpose is to support measurement and reproducibility, rather than to define a separate category based on a particular computational method.

▪ Diagnostic detection and classification

These models assign a diagnostic or morphological category to a region, slide, or case [10]. Examples include tumor subtype classification, grading systems such as Gleason pattern scoring, and distinguishing benign from malignant lesions. By learning visual patterns associated with diagnostic labels, these models support more standardized and reproducible diagnostic decision-making.

▪ Prognostic and therapeutic decision support

These applications estimate prognostic risk, predict molecular biomarkers or treatment response, or otherwise support clinical management decisions [11,12]. Outputs may be categorical labels, continuous scores, or probabilities. Regression is therefore one possible computational formulation rather than a separate clinical function. Clinical usefulness requires validation for the intended population, calibration where applicable, and evidence that the output contributes meaningfully to the relevant decision.

▪ Workflow optimization

These algorithms are designed to support operational efficiency within pathology workflows. Examples include triaging slides that are likely to contain diagnostically significant findings, flagging cases that may benefit from ancillary testing, or prioritizing large case volumes based on predicted urgency. Although they may rely on underlying classification or regression models, their primary aim is to streamline workflow and improve turnaround time.

▪ Exploratory analysis and hypothesis generation

Some AI models can explore large image datasets to identify previously unrecognized morphological relationships or spatial patterns without predefined labels. These unsupervised approaches may help generate new biological or diagnostic hypotheses, though they remain primarily research-oriented and are not used for direct clinical decision-making.

Computational approaches should be distinguished from the functions described above. Supervised, self-supervised, and weakly supervised learning, foundation models, multimodal image–molecular integration, and generative methods can all support several application categories Classification, regression, segmentation, representation learning, and clustering describe computational tasks or methods rather than mutually exclusive clinical functions. Agentic systems may coordinate multistep activities, such as routing cases or drafting reports [13], but do not constitute a separate clinical-function category. Their safety and effectiveness require assessment for the specific workflow and degree of autonomy. Published feasibility, external clinical validation, and documented routine deployment remain distinct levels of evidence. Table 1 therefore summarizes application-specific evidence considerations without assigning a universal readiness label to an entire category.

## 4. Developing AI Algorithms: Basic Requirements and Key Considerations

Multiple critical issues should be considered when developing AI algorithms (summarized in Table 2). But even before the precaution discussion comes to place, the first fundamental question that needs to be addressed is which particular AI algorithm should be prioritized for development. Pathologists address hundreds of diagnostic and analytical questions each day. Yet resources for AI development are limited. There are diagnostic routine tasks that may be very common and still not represent true rate-limiting workflow bottlenecks (e.g., identification of typical tissue structures, such as glands or lymph nodes, or routine benign vs. malignant tissue calling). Instead, rate-limiting tasks typically refer to tasks that require exhaustive manual investigation for relatively sparse events, or complicated cases with high inter-observer variability. Therefore, careful prioritization is essential. Tasks with the greatest sum of clinical utility, frequency, and impact on workflow efficiency and patient outcomes naturally should become the first choice.

The next consideration is whether adequate data of the right kind is available to the team in order to build and validate such a model. Depending on the intended application and model complexity, the size of required datasets can vary greatly. Every project requires a strong training dataset (size and variability) to support robust internal learning. Inadequate or poorly curated datasets often lead to model overfitting and limited potential for generalizability, regardless of AI algorithmic sophistication [14,15]. The next concern is identifying the team of expert pathologists who will lead and sustain the labor-intensive process of manual slide annotation. Pathologists are often heavily burdened with clinical responsibilities, leaving limited time for meticulous slide annotation efforts, which can take weeks or months of work. Recently, modern transfer learning frameworks [16,17] and the introduction of foundation models for computational pathology applications have helped somewhat mitigate these constraints [18]. Complicating things further, the diagnostic standards in pathology (e.g., tumor grading/staging diagnostic definitions) are continually evolving, requiring pathologists and algorithms to keep adapting to updated definitions and criteria [19].

Data quality, selection, and clinical representativeness are critical, but training and clinical evaluation must be distinguished. During training, class weighting, over-sampling, under-sampling, or other balancing strategies may legitimately improve learning from uncommon diagnoses. By contrast, evaluation of clinical usefulness must account for the target population and its disease prevalence: case-enriched test sets can measure discrimination, but predictive values derived from them may not apply directly to routine practice [20,21]. Where risk probabilities are used, calibration should also be assessed in the target population. Spectrum bias may persist when rare entities, subtle presentations, demographic subgroups, or referral patterns are underrepresented [20,22,23,24]. Technical variation in fixation, staining, section preparation, scanning and image management can further compromise transfer between sites. Stain normalization and augmentation can reduce aspects of appearance variation, but cannot correct unrepresentative disease spectra or systematic labeling differences. Federated learning may support multi-center learning without routine central pooling of raw images, but does not guarantee representativeness, privacy, or fairness. Independent external testing across institutions and platforms, subgroup performance analysis, and local prospective evaluation remain necessary (Table 3).

## 5. Validation and Evaluation for Clinical Practice

Analytical validity and clinical performance should be evaluated for the specific intended use: screening or triage, second-read assistance, quality assurance, quantification, or diagnostic support. Relevant endpoints include sensitivity, specificity, predictive values, false-alert burden, calibration when probabilities are reported, and effects on pathologist decisions. For example, in a controlled breast lymph node metastasis reader study, Retamero et al. evaluated 167 whole-slide images with three pathologists and reported mean sensitivity of 81.2% without assistance and 93.2% with AI assistance; the overall difference did not meet conventional statistical significance (*p* = 0.09). Mean review time per slide was reported as 129 versus 58 s, respectively [25]. These observations indicate the potential of AI assistance in a reader-study setting; they do not establish corresponding reductions in end-to-end reporting time, costs, or improved patient outcomes. Evidence from prostate cancer grading investigations similarly requires interpretation according to study design and the chosen comparator [26]. Baseline pathology error estimates vary by specialty, case mix, and how discordance is defined [27]. Any increased sensitivity must also be considered against false-positive findings, additional testing, and possible downstream harms.

Therefore, technical performance should be assessed in a clinically grounded manner, recognizing that statistically significant improvements in performance metrics do not necessarily translate into clinically meaningful benefits or improved patient outcomes, and may contribute to over-testing and over-diagnosis if not thoughtfully integrated into practice [28,29,30]. Once the technical performance aspects of the algorithm are confirmed, appropriate external validation is the next important consideration. Novel algorithms must be validated rigorously to ensure that they are accurate and reliable when implemented on clinically representative test cohorts that match the intended algorithm’s deployment setting. Validation frameworks should include strong evidence that the algorithm generalizes well across different scanners, staining protocols and patient populations. This, however, requires secure communication channels between institutions for image sharing, which can be operationally challenging (the recent literature suggests a few tools for safe and efficient exchange of research and clinical material among institutions [31]). Accordingly, universal adherence to standardized frameworks, such as the Digital Imaging and Communications in Medicine (DICOM) [32], along with standardization of staining and processing protocols are becoming more and more essential. Beyond algorithm-only testing, validation should also include benchmarking against expert pathologists to ensure their clinical performance and utility in real-world workflows. Depending on the specific task and intended application, multiple performance metrics should be selected and transparently reported to accurately reflect the intended clinical use (summarized in Table 4). Consistent with this, the College of American Pathologists (CAP), recommends that validation should be designed to match the specific clinical purpose (e.g., screening/triaging versus ion support versus quality assurance) and the practice environment in which the AI algorithm will be used. CAP has provided some general guidance on validating AI systems in anatomic pathology [33], but it is becoming evident that a ‘one size fits all’ validation list is unlikely to satisfy all applications. Safety evaluation should extend beyond discrimination. Explainability methods such as heatmaps can support error review but must not be mistaken for faithful explanations or proof of validity. Calibration evaluates whether predicted probabilities reflect observed frequencies; uncertainty estimates and out-of-distribution flags may identify cases requiring more intensive human review, provided these mechanisms themselves are validated. Robustness assessments should include staining, scanners, artifacts, image compression, and clinically realistic perturbations, with adversarial testing where the security threat model warrants it. Failure and abstention behavior, thresholds for escalation, and human oversight should be prespecified for the intended application.

An evidence hierarchy is useful when considering adoption. Retrospective model validation tests diagnostic performance on existing data; assisted-reader studies evaluate human–AI interaction under controlled conditions; prospective silent deployment tests technical operation against live workflow without influencing care; and prospective interventional evaluations assess actual effects on turnaround time, workload, decisions, patient outcomes, and adverse consequences. Economic models estimate possible value under their assumptions but do not directly measure savings at other sites. Comparisons should state the cohort, comparator, clinical setting, uncertainty intervals where reported, and endpoints actually measured. Silent trials can assess performance and operational readiness, but they cannot alone establish the effect of presenting AI output on clinical decisions [34].

As briefly mentioned earlier, a fundamental challenge during the clinical evaluation of an AI diagnostics algorithm is how to define an appropriate reference standard (known as “the ground truth”), particularly for diagnostic tasks for which there is already significant inter-observer variability in practice. For every task it can be different, but benchmarking efforts can include consensus calling among various expert pathologists, the additional use of ancillary tests (such as immunohistochemistry or molecular assays), or even outcome-linked endpoints (where available). In any case, the performance of the algorithm should always reflect the intended use of this tool (e.g., triage, second-read, or quantitative assist) instead of just providing an isolated model performance metric. Finally, it should be noted that, when possible, prospective evaluation of the algorithm’s performance is better in establishing true, real-world clinical value, compared to retrospective evaluations (most commonly used). Prospective silent deployment, in which AI outputs are withheld from clinicians and do not alter patient care, can assess diagnostic performance on incoming cases, technical reliability, inference latency, data compatibility, and potential failure modes under routine operating conditions [34]. It can also inform hypotheses about possible workflow benefits. However, it cannot directly establish reductions in pathologists’ workload or reporting time, changes in clinical decisions, or improvements in patient outcomes attributable to AI assistance. These effects require a subsequent user-facing evaluation against an appropriate comparator.

## 6. Implementation in Clinical Practice: Real-World Adoption Considerations

On top of its performance metrics, adoption of a new AI digital pathology tool in practice is largely dependent on how efficiently it can integrate to the existing operational pipelines [35]. Specifically, each new AI tool should be smoothly integrated with the existing viewer, image-management system (IMS) and the laboratory information system (LIS) used by the department. Moreover, especially when real-time, on-premise inference is needed there is requirement for strong computational support (GPUs) devoted exclusively to clinical practice. Careful consideration of possible latency constraints and development of clear policies for managing discordant (pathologist vs. AI) cases are also important. Hence, adoption of each new tool should be considered in a staged fashion (starting with ‘silent’ parallel deployment) that would allow prospective monitoring of all these attributes in real clinical settings.

Beyond integration, the practical value of deployment is largely determined by its impact on time efficiency and cost. AI-supported digital pathology tools can significantly accelerate task completion especially for tedious tasks that require comprehensive evaluations of large tissue areas. For instance, recent studies have shown that the use of automated AI detection algorithms of lymph node metastases resulted in a statistically significant reduction of average review time per image, while maintaining or improving sensitivity [36]. Other examples include AI-based biomarker quantifications, automated definition of regions of interest or AI-based triaging of cases [37]. In all these areas, AI tools have been shown to improve task-completion times. Yet it should be noted that the use of AI tools does not by default guarantee time savings. The potential need for additional verification steps, the risk for introduction of false positives and the emergence of technical issues related to tool integration can all offset timing gains. That is why careful and staged prospective evaluation in real clinical settings is critical.

Cost-effectiveness requires a local, system-level perspective. Du et al. modeled the effectiveness and cost-effectiveness of AI-assisted prostate cancer diagnosis within a Swedish healthcare context [38]; the findings are informative but depend on assumptions about patient pathways, staffing, baseline accuracy, and unit costs and should not be generalized automatically to other laboratories or AI tasks. A comprehensive business case includes procurement or development, software licensing, scanners and image storage, secure computing infrastructure, networking, integration with laboratory information and image-management systems, pathologist annotation and validation, cybersecurity, staff training, quality assurance, ongoing model monitoring, maintenance, and periodic revalidation [39]. Potential gains include reduced review time, fewer repeat reviews, and improved prioritization, but these must be measured against any extra verification, false alerts, and downstream testing. Before adoption, laboratories should define time horizon, perspective, comparator, capital and recurring costs, throughput, and measurable benefits with sensitivity analyses for uncertain assumptions.

Lastly, even well-validated tools can fail in practice if clinicians either over-trust or under-trust their outputs. Overreliance on AI algorithms can lead to “automation bias”, especially for junior pathologists. A diagnosis made by an AI algorithm is often considered to be high-tech and, therefore, superior to the human eye, which is not always the case. As the well-known saying amongst pathologists goes, “tumors don’t read books”: there are a number of gray areas in pathology that the human mind deciphers, considering the whole clinical picture and implications for future treatment. The human mind is able to come to certain decisions and triage the information given based on a rational thought process. Certain aspects of a challenging case are weighed differently than others, and it is difficult to ascertain which of these aspects AI gives more weight to in its decision-making process, which can put the future of pathology in a dangerous position. Practically, the consequences of overreliance include reduced diagnostic accuracy, incorrect treatment plans, reduced pathologist competency, and algorithm aversion. A recent study examined the overreliance on AI algorithms and found that AI-assisted pathologists had a higher accuracy for detecting high-grade dysplasia and invasive carcinoma, and AI improved inter-observer reliability. There were, however, cases of overreliance that have been observed to result in omission of correctly diagnosed invasive cancer that was initially diagnosed using unassisted examination of the slide [40]. This study suggests the integration of confidence scoring and continuous education for pathologists as tools to optimize safe integration of AI into everyday practice.

Evidence from radiology illustrates the potential for AI assistance to improve reader performance in selected settings [41], but these findings cannot be assumed to be generalized to pathology. Human oversight remains an important safeguard, although its presence alone does not prevent automation bias. In pathology, the benefits and risks of combined human–AI interpretation should be evaluated for the specific task, user group, interface, and practice setting, including whether AI assistance introduces new errors or inappropriate reliance.

## 7. A Staged Decision Framework for Clinical Implementation

The proposed framework combines a six-stage implementation pathway with a structured readiness assessment comprising an illustrative multidomain scorecard (Supplementary Table S2). Its intended added value is to make evidence gaps, operational dependencies, and responsibilities explicit when laboratories consider progression between stages. The aggregate score summarizes documented readiness but does not independently authorize progression: unmet critical requirements cannot be offset by higher scores in other domains. The domains and weights can change from institution to institution, and no universal numerical deployment thresholds are proposed. This framework has not been formally validated and does not replace application-specific evidence, local governance, or regulatory requirements. The illustrative scorecard allocates 100 points across ten domains: intended use and clinical need, data quality and provenance, model development transparency, validation and performance, bias and fairness, safety and risk assessment, operational readiness, monitoring and lifecycle management, pathologist acceptance and interpretability, and regulatory and governance considerations. The proposed ratings and weights require evaluation for inter-rater reliability, sensitivity to weighting choices, and usefulness across laboratories and intended uses.

Stage 1—Clinical need and intended use: The first stage involves identifying the patient or workflow problem, target population, current comparator, accountability, and measurable benefit.

Stage 2—Tool selection and evidence appraisal: The second stage involved assessing available products or local algorithms by intended-use fit, evidence maturity, regulatory status, dataset provenance, interoperability, costs, security, and relevant reference standards.

Stage 3—Local validation and readiness: The third stage involved using representative independent cases to test performance, subgroups, robustness and failure modes and confirming computational capacity, integration, privacy, escalation pathways and clinician training.

Stage 4—Prospective pilot: The fourth stage involves considering silent parallel deployment first to assess diagnostic performance, technical reliability, latency, and operational compatibility without influencing care. If appropriate safety and readiness criteria are met, a controlled user-facing evaluation should follow to assess effects on workload, turnaround time, reader behavior, clinical decisions, adverse consequences, and implementation costs against predefined endpoints and an appropriate comparator.

Stage 5—Controlled clinical launch: The fifth stage involves deploying only after agreed thresholds are met, appropriate regulatory and institutional requirements are satisfied, and named clinical and technical owners approve.

Stage 6—Monitoring, update or withdrawal: The sixth stage involves monitoring diagnostic and operational metrics, drift, calibration and subgroups over time and predefining investigation and stop rules, retraining and revalidation criteria, version approvals, rollback, and conditions for discontinuation.

Suggested go/no-go criteria include minimum task-specific diagnostic performance, acceptable false-positive and false-negative burdens, feasible inference latency, clinically meaningful improvement versus the existing workflow, sustainable costs, and an assigned oversight plan. These criteria should be defined locally, rather than replaced by a universal numerical cutoff.

## 8. Lifecycle Management: Performance Drift, Monitoring, and Updates

The training process does not end once the algorithm has been developed. Clinical deployment requires lifecycle management, because data and practice environments can change. Temporal performance degradation is a potential risk rather than a universal or inevitable event [42]. Vela et al. investigated 128 model–dataset combinations across multiple domains and found degradation in 91% of those combinations; this figure is not an estimate of the proportion of pathology AI tools that will degrade [42]. Departments should monitor relevant input characteristics and prespecified performance metrics, including clinically important errors, subgroup outcomes, calibration where appropriate, latency and availability. Defined warning and stop thresholds should trigger investigation, escalation, temporary suspension when safety is threatened, or a supervised update. Scanner, staining, workflow, software, and model changes require impact assessment and, where warranted, revalidation before reactivation. Each update should have documented version control, responsible approval, audit trail, and a rollback plan. Retraining is an option after root-cause investigation, not an automatic response to every shift.

## 9. Regulation and Oversight

Regulatory requirements depend on jurisdiction, intended use, risk classification, and deployment model. In the United States, AI-enabled software meeting the definition of a medical device falls under applicable US Food and Drug Administration (FDA) medical device pathways, subject to the relevant risk-based requirements [43,44,45,46]. In the European Union, software qualifying as a medical device or an in vitro diagnostic medical device follows the applicable Medical Device Regulation (MDR) or In Vitro Diagnostic Medical Device Regulation (IVDR), including relevant conformity assessment and CE-marking obligations. The European Medicines Agency (EMA) is not the general authority that authorizes clinical pathology AI software as medical devices. A tool labeled for research use only should not be treated as clinically authorized; rules for laboratory-developed applications differ among jurisdictions. Regulatory status, permitted uses, required human supervision and change-control responsibilities should be established before clinical deployment.

The distinction between systems that inform decisions and those that drive clinical management can affect risk assessment, but terms such as “decision support” do not alone determine regulatory status. The actual intended purpose, output, and degree to which clinicians can independently review the basis for a recommendation are important. Regulatory documentation should cover analytical and clinical evidence, software and cybersecurity controls, risk management, change management and post-market oversight as applicable. The laboratory remains responsible for assessing whether a given authorization or conformity assessment covers its intended clinical workflow [45,46].

Pathology AI also depends on the complete digital workflow: scanner, viewer, image-management system, laboratory information system, network, storage and algorithm. Compatibility and validation at these interfaces are essential, although different components may fall under different regulatory obligations [47]. Professional and multi-stakeholder initiatives, including the Coalition for Health AI and the Health Artificial Intelligence Validation Network, provide resources that can inform responsible evaluation; these initiatives should not be confused with statutory regulatory authorities [48,49].

## 10. Conclusions

AI systems offer promising opportunities to improve selected diagnostic and operational activities in anatomical pathology, but evidence remains heterogeneous across indications, study designs, and deployment settings. A technically accurate algorithm is not necessarily clinically useful or economically sustainable. We therefore advocate a staged, decision-oriented pathway in which meaningful clinical needs are identified first, tools are assessed against appropriate evidence and local constraints, implementation is evaluated prospectively, and clinical performance is monitored throughout the lifecycle. Foundation models and multimodal systems may broaden future applications, but their introduction likewise requires fit-for-purpose validation and governance. Rather than assuming that AI will inevitably become an operational necessity in every laboratory, its role should be determined by measurable benefit, patient safety, affordability, and institutional readiness.

## Figures and Tables

**Table 1:** Functional applications of pathology AI, representative examples, and evidence considerations. Exploratory applications are primarily research-oriented. Readiness varies by product and intended use; examples do not imply regulatory authorization or routine adoption.

Function or Research Purpose	Representative Applications	Clinical Evidence and Readiness
Image analysis and quantification	Tumor region detection, IHC scoring, mitotic counting, fibrosis burden estimation, tumor cellularity assessment	Analytical accuracy, reproducibility, and local validation. Selected applications have reached clinical implementation.
Diagnostic detection and classification	Lymph node metastasis detection, cancer grading, tumor subtype classification	Independent diagnostic validation, pathologist reader studies, and assessment of clinical performance. Implementation readiness varies by application.
Prognostic and therapeutic decision support	Prognostic modeling, molecular biomarker prediction, treatment response prediction	External validation, calibration where applicable, and demonstration of clinical utility. Many applications remain investigational.
Workflow optimization	Case triaging and prioritization, quality control (QC), automated reporting assistance	Prospective evaluation of workflow efficiency, turnaround time, patient safety, and human oversight. Clinical adoption depends on local implementation conditions.
Exploratory analysis and hypothesis generation	Spatial pattern discovery, identification of novel morphological features, generation of biological or diagnostic hypotheses	Primarily research-oriented and hypothesis-generating. Independent biological and clinical validation is generally required before diagnostic use.

**Table 2:** Key considerations for implementation of machine-learning models in pathology.

Access to large, diverse, and well-curated datasets: Sufficient data for training. Datasets for internal validation, and independent external evaluation (ideally spanning institutions, scanners, staining protocols, and patient populations to ensure generalizability). Annotation burden and ground-truth uncertainty: Manual annotation by expert pathologists (costly, time-intensive, inter-observer variability). ML frameworks (weakly supervised learning, self-supervised learning, transfer learning, etc.). Bias risks: Demographics. Tissue processing protocols. Institutional practice patterns. Secure data sharing and privacy preservation: Multi-institutional collaboration requires robust frameworks for data governance, de-identification, secure transfer, and compliance with local and international privacy regulations. Prospective performance validation: Models must be evaluated on truly independent datasets. Monitor model performance over time for performance degradation. Regulatory and lifecycle considerations: Implementation must address strict regulatory approval and auditability.

**Table 3:** Potential approaches to mitigate and evaluate dataset bias in artificial intelligence applications in digital pathology [20,21,22,23]. No single method guarantees generalizability.

No.	Approach	Recommended Considerations
1	Standardization of laboratory procedures	Standardize and document tissue handling, staining protocols, and scanner acquisition procedures.
2	Image normalization and augmentation	Consider color normalization and data augmentation to address technical variability, while independently verifying that clinically relevant information is preserved.
3	Dataset diversity and external validation	Ensure patient-level data separation and use independent external test cohorts representing multiple institutions, scanners, and patient populations.
4	Assessment of population bias	Evaluate subgroup performance, disease spectrum, prevalence-dependent predictive values, and model calibration in the intended clinical population.
5	Prospective clinical evaluation	Use representative prospective local cohorts to assess model performance within the clinical workflow in which the algorithm will be deployed.
6	Federated learning and data governance	Consider federated learning under appropriate privacy and data governance frameworks, recognizing that data heterogeneity and selection bias still require evaluation.
7	Performance metrics and evaluation	Predefine appropriate discrimination, calibration, and operational safety metrics according to the intended clinical application.
8	Transparency and human oversight	Document remaining limitations, predictive uncertainty, and appropriate pathways for human review of uncertain or discordant cases.

**Table 4:** Key factors to consider when assessing the clinical usefulness of AI tools.

Technical performance: Evaluation of model performance using metrics such as sensitivity, specificity, and predictive values. Assessment of workflow efficiency and turnaround time: Evaluation of whether the model significantly reduces time to diagnosis, improves the workload, and whether it integrates seamlessly into existing clinical workflows. Clinical utility and decision impact: Assessment as to whether the model impacts clinical decisions and improves diagnostic confidence and patient management. Cost-effectiveness and resource utilization: Balancing the cost of implementation and maintenance (including infrastructure, validation and personnel) with measurable gains in time efficiency, sensitivity and specificity, and patient outcomes.

## Data Availability

No new data were created or analyzed in this study. Data sharing is not applicable to this article.

## Supplementary Materials

The following supporting information can be downloaded at https://www.mdpi.com/article/10.3390/diagnostics16213653/s1: Table S1: Published foundational models in pathology. Adapted from Ochi et al. [8]; Table S2: Proposed readiness scorecard and mandatory stage-specific gates for pathology AI implementation.

## Abbreviations

AI	Artificial intelligence
GPU	Graphics Processing Unit
QC	quality control
IHC	immunohistochemistry
LVI	lympho-vascular invasion
HP	Helicobacter pylori
LN	lymph node
WSI	whole-slide image
DICOM	Digital Imaging and Communications in Medicine
FDA	Food and Drug Administration
LDT	Lab Developed Test
QA	Quality Assurance
CHAI	Coalition for Health AI
HAIVN	Health and Artificial Intelligence Validation Network
ML	Machine Learning
DP	Digital Pathology
