# Supplementary material for: High Time for Pathology AI: Practical Considerations for Turning Potential into Practice
Source: Diagnostics (Basel). 2026 Nov 5;16(21):3653. doi: 10.3390/diagnostics16213653 (PMC13763417; doi:10.3390/diagnostics16213653)
Supplement: Supplementary file 1 [file diagnostics-16-03653-s001.zip › diagnostics-4649613-supplementary.pdf]

Supplementary Table S1. Published foundational models in pathology

Foundational Model	Pretraining dataset used	Number of WSIs used
CTransPath	TCGA, PAIP	32,220
Lunit	TCGA, TULIP	36,666
Phikon	PanCancer40M, TCGA	6,534
PLIP	OpenPath	Unspecified
REMEDS	JFT-300M, CAMELYON16, TCGA, slides from individual institution	20,018
Virchow	Dataset from individual institution	1,488,550
UNI	Mass-100K, Mass 1K, Mass 22K	100,426
CONCH	Online educational sources	Unspecified
PRISM	Dataset from individual institution	587,196
Prov-GigPath	Dataset from individual institution	171,189
TANGLE	TCGA, TG-GATEs	8,671
RudolphV	Datasets from multiple institutions/laboratories	133,998

*Table adapted from Ochi et. al [8]

Supplementary Table S2. Proposed readiness scorecard and mandatory stage-specific gates for pathology AI implementation

A. Provisional domain scorecard

Domain	Maximum points	Illustrative evidence to document
Intended use and clinical need	10	Document the clinical or workflow problem, target population, comparator, intended users, accountable owner, and measurable benefit.
Data quality and provenance	10	Document data sources, permissions, patient-level separation, reference-standard procedures, relevant technical variation, and representativeness for the intended setting.
Model development transparency	10	Document the tool/version, available development and evaluation information, preprocessing, known limitations, and any foundation-model dependencies
Validation and performance	10	Document independent evaluation appropriate to the stage, local performance, uncertainty, relevant comparators and endpoints, failure modes, and calibration where applicable. Distinguish silent testing from user-facing evidence.
Bias and fairness	10	Assess relevant population subgroups, disease spectrum, prevalence-dependent performance, and technical or institutional sources of bias. Document limitations and mitigation.
Safety and risk assessment	10	Document clinically important false-positive and false-negative harms, escalation and abstention procedures, human oversight, incident response, and stage-appropriate safeguards.
Operational readiness	10	Assess scanner/viewer/IMS/LIS compatibility, compute, latency, reliability, staffing, training, and sustainable capital and recurring costs.
Monitoring and lifecycle management	10	Document named monitoring owners, metrics, investigation and stop rules, update approval, revalidation, version control, rollback, and withdrawal procedures.
Pathologist acceptance and interpretability	10	Document representative user involvement, training, output comprehension, review of uncertain or discordant cases, and evaluation of inappropriate reliance during user-facing assessment.
Regulatory and governance considerations	10	Document applicable regulatory and institutional requirements, permitted use, privacy/security review, accountability, and approvals required for the next stage.
